# Fear Appeals in Anti-Knife Carrying Campaigns: Successful or Counter-Productive?

**DOI:** 10.1177/08862605211064237

**Published:** 2022-01-17

**Authors:** Zoë Hobson, Julia A. Yesberg, Ben Bradford

**Affiliations:** 1 Department of Security and Crime Science, Institute for Global City Policing, 4919 University College London, London, UK

**Keywords:** knife crime, fear appeals, media campaigns, terror management theory, mortality salience

## Abstract

In the UK, knife crime continues to be a persistent and worrying concern. Media campaigns are often used by police and anti-knife crime organisations in an attempt to discourage young people from picking up a weapon. Many focus on the potentially devastating consequences associated with carrying a weapon, with the aim of provoking fear and thus a deterrent effect. In this paper, we present the findings from two experimental studies exploring the effects of exposure to fear-based knife crime media campaigns on young people’s intentions to engage in knife-carrying behaviour. Utilising a terror management theory perspective, in both studies we found that exposure to knife-related campaign imagery increased mortality salience, but there was no effect of campaign condition on willingness to carry a knife or on perceived benefits of knife-carrying. Although knife-related self-esteem/cultural worldviews predicted attitudes towards knife-carrying, such views did not moderate the effect of exposure to knife-related campaign imagery, and there was no effect of priming participants’ to consider the value of behaving responsibly. Implications and suggestions for future research are discussed.

Knife crime is currently a significant issue in the UK: legally, politically and socially. Media outlets describe a ‘knife crime epidemic’ engulfing society (The Independent, 2020). Although this assertion contains a strong element of hyperbole, it is nevertheless the case that in England and Wales in 2019, 45,627 offences involving knives or sharp instruments were recorded by police – a record high – with a 7% rise year-on-year, and a 49% increase since 2011 when comparable records began (Office for National Statistics, 2020). The data reveals that 25% of people convicted for a knife offence were men aged 18–24 years (Office for National Statistics, 2020). Knife crime also appears worryingly prevalent amongst young people, with 4,562 young people aged 10–17 years sentenced for carrying a knife or offensive weapon in England and Wales in the year ending September 2019. This figure is the highest ever recorded and rose by 1.5% on the previous 12 month period (Office for National Statistics, 2020).

Despite the scale of the issue, there has been little systematic examination of the factors that influence knife-carrying (Palasinski et al., 2021). Commonly cited correlates of such behaviour include fear of crime and violence, need for protection, desire for social status, previous experience of violent victimisation, peer criminality and distrust of authorities (Bégue et al., 2016; Brennan, 2019; McVie, 2010; Palasinski & Riggs, 2012). A recent longitudinal analysis of a sample of 10–25 year olds in England and Wales found that weapon-carrying was predicted more by previous experience of violence and crimogenic factors than fear of victimisation (Brennan, 2020). Yet, despite this lack of clarity, anti-knife crime media campaigns used by police, government, and other organisations typically focus on fear and the mortality-related risks of carrying knives (e.g. the potential for people to be seriously injured or killed; Childline, 2020). These campaigns are often accompanied by images of knives and/or the aftermath of a stabbing, with the rationale being that exposure to such information will provoke fear and deter people from carrying out such behaviour.

While a rational choice theory perspective might suggest such an approach would be effective (i.e. by causing people to consider that the costs of carrying a knife outweigh the benefits), there has been very little research on the efficacy, or otherwise, of such efforts, meaning that sometimes extremely disturbing images are disseminated with no real understanding of the effect they have on those exposed to them. Further, there is a strong theoretical rationale – provided by Terror Management Theory (TMT) (Greenberg et al., 1997) – for predicting that fear-based media campaigns focussing on mortality-related risks may actually have a backfire effect, leading to an increase in the targeted behaviour.

This paper seeks to understand whether fear appeals in knife crime media campaigns are likely to be successful or counterproductive. We draw on TMT, presenting the findings from two experimental studies designed to explore the effects of providing information to young people about the mortality-related risks of knife-carrying. The paper proceeds as follows. First, we outline the evidence base for the effectiveness of fear appeals in public health and crime-related contexts. Second, we outline the TMT perspective and how it could be applied to understanding the potential impact of fear-based knife crime media campaigns. We then present separate methods, results and discussions for the two studies before discussing the implications and conclusions from our findings.

## Fear Appeals in Anti-knife Carrying Media Campaigns

Fear-based media campaigns have been used extensively in various public health fields in attempts to alter a range of behaviours, including alcohol and tobacco use, illicit drug use, heart disease prevention and sex-related behaviours (Tannenbaum et al., 2015; Wakefield et al., 2010). These campaigns typically attempt to arouse fear in people by emphasising the potential danger, harm or risks associated with the targeted behaviour (e.g. that smoking tobacco causes lung cancer). Often the campaigns are accompanied by graphic images depicting the consequences of the target behaviour. There are differing views on whether fear-based messages are effective or counterproductive (e.g. Ruiter et al., 2014), but a meta-analysis of the literature on fear-based campaigns found that they *can* be effective at positively changing people’s attitudes, intentions and behaviour when the message includes statements about efficacy, is high in depicted susceptibility and severity, and targets a one-time behaviour rather than a repeat behaviour (Tannenbaum et al., 2015).

Fear-based appeals are frequently used in anti-knife carrying media campaigns. For example, recent campaigns in the UK – including ‘Lives not Knives’ (Leicestershire Police, 2020) and ‘#Lifeorknife’ (West Midlands Police, 2019) highlight the mortal danger of carrying a knife, with the aim of provoking fear and ultimately deterring young people from engaging in this behaviour. However, there is little evidence on the efficacy of these efforts. Particularly salient in the current context is that studies on fear appeals in areas relating to crime and offending have shown them to be less effective, and even counterproductive, in young males (Lennon et al., 2010; Taubman Ben-Ari et al., 2000). For example, in a driving simulator experiment young men drove faster than a control group after having seen a frightening film about road safety (Taubman Ben-Ari et al., 2000). In another study, young male participants reported greater intentions of engaging in distracted driving behaviours after viewing social marketing fear appeals to do the opposite (Lennon et al., 2010). Other related research has also shown that interventions specifically aimed at provoking fear of the consequences of offending in young people may have backfire effects. The (in)famous ‘Scared Straight’ programmes, which aimed, through prison visits and other means, to provoke fear of imprisonment among young people in order to deter them from committing crime have been found not only to fail in terms of deterrence but to actually increase offending behaviour (Petrosino et al., 2013).

By contrast, Palasinki et al. (2021) recently conducted a series of studies exploring the effect of exposure to different anti-knife carrying slogans and posters on knife-carrying tolerance among adult males aged 18–25 years. They found that injury-related slogans and posters – those emphasising physical trauma – were the most persuasive types of messaging, compared to those emphasising pathology (i.e. deviance), respect, control or masculinity. Interestingly, injury-related messaging was rated as more persuasive than death-related messaging, suggesting there may be a ‘threshold’ after which fear appeals become less effective. Palasinski et al. (2021) attributed their findings to protection motivation theory (Maddux & Rogers, 1983) – the idea that motivations for self-protection are driven by threat appraisal and coping appraisal.

Protection motivation theory suggests that people confronted with the choice to enage in ‘risky’ behaviour – for example carry a knife – weigh up the perceived costs of reducing this risk (by not carrying a knife) against the perceived benefits of carrying it (Palasinski et al. 2021). This research therefore resonates with a broadly rational choice perspective that, as noted above, would suggest that exposure to images relating to injury and/or death will dampen intentions to carry a knife because they highlight the potential costs involved in doing so. This is, we assume, the motivation behind many of the anti-knife crime campaigns of recent years. Police and others are aware, that is, that young people carry knives because they fear for their own safety and/or gain value in other ways from doing so (Brennan, 2019). Making them more aware of the dangers inherent in such actovotu could therefore shift their calculus of risk, and of costs and benefits, making them less likely to engage in this behaviour.

In sum, the research on fear-based media campaigns is mixed (Tannenbaum et al., 2015). Palasinski et al. (2021) found that injury-related messaging was persuasive in a sample of young adult males, but their study measured tolerance towards knife-carrying (e.g. whether it could be seen as acceptable and justified), rather than intentions or behaviours surrounding knife crime. Other studies have shown fear appeals tp be less effective, and even have the ability to backfire, in young males (Lennon et al., 2010; Taubman Ben-Ari et al., 2000). TMT offers an account of why this might be the case.

## Terror Management Theory

TMT is a social and evolutionary psychology theory which posits that human beings are aware of the inevitability of their own death (Greenberg & Arndt, 2011). The theory contends that reminding people of their mortality brings about a severe feeling of threat and fear, and the immediate response – their ‘proximal defence’ – is often to deny their own vulnerability and actively suppress such thoughts (Ivanov & Vogel, 2017; Pyszczynski et al., 1999). Yet, as the salience of their mortality subsides and moves away from their focal attention, people are likely to activate ‘distal defences’ in order to protect themselves and preserve a positive sense of self (Jessop et al., 2008; Ivanov & Vogel, 2017).

Distal defences are unrelated to death but ‘imbue one’s life with meaning, value, and the promise of either literal or symbolic immortality’ (Pyszczynski et al. 2021, p.175). *Cultural worldviews* and *self-esteem* are examples of distal defences which play important parts in people’s attempts to maintain psychological equilibrium. Cultural worldviews are socially constructed and shared beliefs about the nature of reality from which people obtain a sense of meaning, value and permanence (Arrowood & Cox, 2020). Identifying with a group, religion, friends or family can provide, literally or symbolically, a sense of immortality. If people abide by the norms of these groups, then their self-esteem will be bolstered, since such adherence indicates group membership and standing. It is important to note that this process may occur in relation to ‘sub-cultural’ groups and norms. For example, if one identifies with a group that values perceived strength from carrying a weapon, then one should feel good about oneself when following this norm.

TMT posits that people manage the anxiety-inducing awareness of the inevitability of death through maintaining faith in their cultural worldviews and self-esteem (Pyszczynski et al., 2015). Research has indicated that when mortality is made salient, people express greater support for others who accept their cultural worldviews, whilst showing negativity towards those who counter their beliefs (Burke et al., 2010; Greenberg et al., 1992; Pyszczynski et al., 2015). Further, one strategy to shield against mortality-related concerns is to strive for higher self-esteem, which is achieved through actions that align to one’s perceived self-worth and/or subscription to one’s worldviews (Greenberg et al., 1992; Pyszczynski et al., 1999).

Importantly, this strategy extends to risky behaviour. In other words, if an individual’s self-worth and cultural worldviews are associated with risky behaviours, being made aware of their own mortality would lead to *higher* intentions to engage in such behaviour (once mortality salience moves outside the focal attention). Taubman Ben-Ari et al. (1999) looked at the effects of mortality salience on risk-taking whilst driving, finding that high behaviour-specific self-esteem made people *more* likely to drive dangerously after having their mortality made salient to them. Jessop et al. (2008) also demonstrated that young drivers who perceived driving fast as beneficial to their own self-esteem reported higher intentions to take driving risks following exposure to death-related messages. Importantly, the same effect (i.e. higher intentions to take driving risks) was not shown for people with low behaviour-specific self-esteem, suggesting self-esteem is an important moderator variable in understanding the effects of exposure to mortality-related information.

Applying TMT to the current paper, the research outlined above would suggest that exposing the mortality-related risks of knives to young people whose self-esteem and cultural worldviews are strongly linked to knife-carrying may actually make them more likely to engage in knife-carrying behaviour by strengthening commitment to their sub-cultural and group norms. As such, fear-based media campaigns that emphasise the mortality-related risks of carrying knives may be at risk of backfire effects.

### Negating Mortality Salience by Behaving Responsibly

Although research suggests that if risky behaviours are beneficial to people’s self-esteem, they will be more likely to engage in those behaviours when their mortality is made salient, some studies have shown that priming people to consider the value of behaving responsibly could negate these effects. For example, Greenberg et al. (1992) found that when male drivers received a prime to behave responsibly, the effects of mortality salience on taking driving risks were negated. Jessop et al. (2008) found identical results in their study with young male drivers: priming people to behave responsibly increased accessibility of responsibility-related constructs and reduced accessibility of mortality-related constructs, thereby eliminating the effect of mortality salience on intentions to take driving risks.

### The Current Paper

Provoking fear and making people consider their own mortality appears to be a key part of police and anti-knife crime organisations’ communication strategy in attempting to prevent young people from carrying knives. Yet there is currently little research looking at the effect of making people more aware of their own mortality through knife-carrying campaigns. Moreover, TMT suggests that such an approach may be counterproductive for those who have high knife-related self-esteem/cultural worldviews – arguably the types of people who are the target of these campaigns. The current paper aims to explore whether exposure to fear-based anti-knife carrying campaign imagery increases mortality salience and the knock-on effects to young people’s attitudes and beliefs about knife-carrying. Doing so will provide valuable information to any future anti-knife crime strategy.

The paper consists of two studies, which were approved by the ethical review board at University College London (17987/001 and 17987/002). Study 1 focuses on young adult males (18–25 years) in the general population, reflecting those who are most likely to be involved in offences involving a knife (MOPAC, 2018). However, it is clear knife crime is also prevalent in younger people. Study 2 focuses on a smaller sample of participants aged 14–18 years recruited through youth offending services, diversionary schemes, secondary schools, and uniformed cadets (e.g. the police cadets).^1^

Restricting ourselves to behavioural intentions, we test whether exposing young males to mortality-related risks related to knife-carrying affects their willingness to carry a knife in the future and whether they perceive more or less benefits to knife-carrying. Further, we explore the moderating effect of behaviour-specific self-esteem and cultural worldviews. Finally, we prime some participants to think about the value of behaving responsibly for themselves or to others (e.g. family members). If this primed responsibility negates the effect of mortality salience on intention to carry a knife, police and other organisations may be able to design more successful communication strategies.

We hypothesise that:

H1: Viewing fear-based anti-knife carrying campaign images will increase mortality salience.
H2: Viewing fear-based anti-knife carrying campaign images will shift respondents’ willingness to carry a knife and perceived benefits of knife-carrying. There are actually two mutually incompatible hypotheses here. H2a proposes, in line with the intention of the original anti-knife crime campaigns included in the study, that viewing knife-based images will *decrease* willingness to carry a knife and perceived benefits of knife-carrying. H2b draws on TMT to suggest that viewing knife-based images will *increase* willingness to carry a knife and perceived benefits of knife-carrying, but only for participants who have high levels of knife-related self-esteem/cultural worldviews (i.e. knife-related self-esteem/cultural worldviews will moderate the relationship between campaign condition and willingness to carry a knife/perceived benefits of knife-carrying).
H3: Priming participants to consider the consequences of knife-carrying on themselves and others will negate the effects of mortality salience on willingness to carry a knife/perceived benefits of knife-carrying.

## Study 1: Method

### Participants

We recruited 479 young adult male residents of the UK to participate in the study through the online platform Prolific Academic on 23 April 2020. Participants were aged between 18 and 25 years old, with an even spread across all eight age points (ranging from 9% to 17%). Three quarters of participants reported their ethnic group to be White - British, White - Irish or any other white background (75.6%), 12.9% were Asian or Asian British, 4.6% were Black or Black British, 4.4% were Mixed and 2.5% were other. There were no significant differences in demographics across the experimental conditions. In line with Prolific recruitment protocols, participants were paid £0.77 (£5.37/h) for taking part in the study.

### Procedure

We used the online software platform Qualtrics to build and host the experiment. The experiment used a 2 (Campaign imagery: knife-related vs. control) × 3 (Message prime: likelihood of death vs. primed responsibility vs. control) between-subjects design. All study materials are included in the Supplemental Appendix.

First, participants were randomly allocated to one of two campaign imagery conditions. They were presented with four screenshots taken from Twitter that were either:

1. Knife-related – tweets relating to anti-knife carrying campaigns, and, in particular, the message that carrying a knife increases the risk of being stabbed/killed yourself (i.e. a fear appeal)
2. Control – tweets that were unrelated to knife crime and reflected a variety of current media campaigns (e.g. sugary drinks, cybercrime, vehicle tax and Blue Cross charity)

Participants viewed the four tweets sequentially. The order of the tweets was randomised to control for order effects. After viewing all four tweets, participants were randomly allocated to one of three message prime conditions. Here, participants viewed a message designed to look like a government/anti-knife organisation advert that carried a specific message:

1. Likelihood of death – ‘carrying a knife can result in your own death – you are 3 times more likely to be stabbed if you go out carrying a knife’
2. Primed responsibility – “carrying a knife can have devastating consequences on your friends and family – no parent or grandparent would ever want to see their child get injured or be killed”
3. Control – no message

A simple filler task then provided a short delay to remove the knife imagery from respondents’ focal attention. As a manipulation check to test that mortality was indeed salient after the campaign imagery (and to answer H1), participants then completed an ‘accessibility to death’ (Webber et al., 2015) related concepts task (see below).

Participants were then asked a series of questions tapping into their knife-related self-esteem and cultural worldview. Next, participants were next asked a series of questions about their willingness to carry a knife, perceived benefits of carrying a knife, and experiences of knife crime. Finally, they were presented with a further short filler task to act as a distraction from the content of the campaign images (to help destress participants from any negative effects if they had viewed the knife-related imagery) and provided with a full debrief.

## Measures

### Accessibility of Death-Related Concepts

To measure mortality salience we used an implicit test derived from Webber and colleagues (2015). Participants were presented with 20 word fragments and were asked to complete the fragments with the first word that came to mind. Five target words were present in the task (Buried, Coffin, Dead, Killed and Skull). A score of 1 was assigned for every target word that was ‘correctly’ identified. These scores were then summed together for each participant.

### Knife-Related Self-Esteem/Cultural Worldview

To measure knife carrying self-esteem and cultural worldviews, participants rated on a 5-point Likert scale (where 1 = Strongly disagree and 5 = Strongly agree) their agreement with four statements about their perceptions of certain behaviours for their own self-esteem (e.g. Carrying a knife would make me feel protected when in public) and four statements measuring the extent to which carrying a knife was endorsed by their cultural worldview (e.g. My friends would have a higher opinion of me if I carried a knife). Higher scores indicate greater self-esteem and cultural worldviews related to knife carrying. The eight items formed a scale with high reliability (α = 0.84).

### Willingness to Carry a Knife

To measure general willingness to carry a knife, participants were presented with a set of three independent statements (e.g. *‘I would consider carrying a knife when I leave the house’*) and were asked to rate how much they agreed with each statement on a 5-point Likert scale (where 1 = Strongly disagree and 5 = Strongly agree). Higher scores indicate greater willingness to carry a knife. The three items formed a scale with acceptable reliability (α = 0.59).

### Perceived Benefits of Knife-Carrying

To measure participants’ perceived benefits of knife-carrying, participants were presented with six scenarios, three of which were related to risk to self and three were related to risk to others. Participants were either asked how safe they would feel in that scenario if they had a knife (where 1 = Much less safe and 5 = Much more safe) or how likely they would be to carry a knife in such circumstances (where 1 = Much less likely to carry a knife and 5 = Much more likely to carry a knife). It is worth noting that this scale is essentially bi-polar: high scores indicate knife carrying has a positive valence (more likely and increases safety), while low scores indicate knife carrying has a negative valence (less likely and diminishes safety). The aim of the images used in this study was, of course, to move people towards the latter. The six items form a scale with high reliability (α = 0.84).

Confirmatory factor analysis in the package MPlus 7.11 was used to derive and validate latent variables for analysis (knife-related self-esteem/cultural worldview, willingness to carry a knife and perceived benefits of knife-carrying). All observed indicators were set to ordinal, and full information maximum likelihood estimation was used (see Appendix A for a list of the items used, factor loadings and model fit).

### Experience of Knife Crime

As an initial matter, we consider exposure to knife crime within our sample. If participants have previously experienced a knife-related incident, whether as a victim, perpetrator or bystander, then this may influence their reaction to the knife-related campaign imagery and subsequent questions. Responses indicated that, while few respondents had been actively involved in knife crime as a victim (5% of sample), or perpetrator (1% indicated they had committed a crime with a knife), a significant number had been exposed via family, friends or acquaintances (32%). These numbers generally align with data from other sources. Some 2% of adults (aged over 16) were victims of violent crime in 2019/20 (Office for National Statistics 2021), rising to 4% in the 16–24 age group, with men more likely to become victims than women. Coid et al. (2021) report results from a nationally representative sample of men aged 18–34 living in England, Scotland and Wales, collected in 2011, that found 5.5% reported carrying a knife in the past 5 years (although it is not known whether they felt they were committing a crime when doing so). In the current study, there were no significant differences across the experimental conditions in participants’ experiences of knife crime.

## Study 1: Results

### Mortality Salience

An independent samples t-test revealed that our mortality salience manipulation was successful. Consistent with **H1**, participants in the knife-related campaign condition completed significantly more word fragments with death-related words (M = 1.62, *SD* = 0.95) than those in the control condition (M = 1.43, *SD* = 0.85), *t* (477) = −2.20, *p* = .028.

### Perceptions of Knife-Carrying

To test **H2** and **H3**, a series of linear regression models were used to determine (a) whether campaign condition influenced participants’ willingness to carry a knife/perceived benefits of knife-carrying; (b) whether knife-related self-esteem/cultural worldviews *moderated* the effects of campaign condition on willingness to carry a knife/perceived benefits of knife-carrying; and (c) the influence of priming people to consider the consequences of knife-carrying on themselves and others. Campaign condition (dummy coded 1 = knife-related, 0 = control) was entered as the explanatory variable in Model 1. In Model 2, knife-related self-esteem/cultural worldview scores were added, and an interaction term between campaign condition and self-esteem was entered in Model 3. Finally, in Model 4, a three-way interaction was tested between campaign condition, message prime condition and self-esteem/cultural worldview scores.

As shown in Table 1, inconsistent with **H2a**, there was no significant effect of campaign condition on either willingness to carry a knife (B = 0.01, *p* = .938) or perceived benefits of knife-carrying (B = -0.05, *p* = .385). Viewing the knife-related campaign images did not shift participants’ attitudes towards knife carrying. Controlling for campaign condition, knife-related self-esteem scores were significantly and strongly related to both willingness to carry a knife (B = 0.82, *p* < .001) and perceived benefits of knife-carrying (B = 0.66, *p* < .001). Participants who had high levels of knife-related self-esteem and cultural worldviews were both more likely to state they would be willing to carry a knife and could perceive more benefits to doing so. There was no significant interaction between campaign condition and knife-related self-esteem/cultural worldviews (willingness to carry a knife B = -0.02, *p* = .684; perceived benefits of knife-carrying B = 0.05, *p* = .425). Inconsistent with **H2b**, knife-related self-esteem/cultural worldviews did not moderate the effect of campaign condition on willingness to carry a knife/perceived benefits of knife-carrying.

**Table 1.** Hierarchical multiple regression analysis predicting willingness to carry a knife and perceived benefits of knife-carrying.

	Willingness to Carry a Knife								Perceived Benefits of Knife-Carrying							
	Model 1		Model 2		Model 3		Model 4		Model 1		Model 2		Model 3		Model 4	
	b	S.E.	b	S.E.	b	S.E.	b	S.E.	b	S.E.	b	S.E.	B	S.E.	B	S.E.
Study 1 (***N*** = 479)																
Image condition (ref: control)																
Knife-related	.01	.07	.06	.04	.06	.04	.05	.07	−.05	.06	−.01	.04	−.01	.04	-.02	.08
Self-esteem/cultural worldview			.82***	.03	.83***	.04	.81***	.06			**.**66***	.03	.63***	.04	.67***	.07
Image condition*Self-esteem					-.02	.05	-.07	.08					.05	.06	−.02	.10
Message prime condition (ref: control)																
Likelihood of death							.05	.07							−.02	.08
Primed responsibility							.06	.07							−.02	.08
Image condition*Message prime condition*Self-esteem (ref: control)																
Knife-related*Likelihood of death							.15	.13							.09	.16
Knife-related*Primed responsibility							−.01	.12							.15	.15
R^2^	.00		.67		.67		.68		.00		.48		.48		.49	
Study 2 (N = 57)																
Image condition (ref: control)																
Knife-related	−.14	.21	.11	.20	.01	.46	.21	.73	−.32	.26	.14	.20	−.05	.45	−.07	.71
Self-esteem/cultural worldview			.41***	.11	.39**	.12	.53*	.21			.74***	.11	**.**72***	.12	.80***	.20
Image condition*Self-esteem					.06	.26	−.15	.45					.12	.25	.06	.44
Message prime condition (ref: control)																
Likelihood of death							−.28	.71							.82	.69
Primed responsibility							−.01	.73							.73	.71
Image condition*Message prime condition*Self-esteem (ref: control)																
Knife-related*Likelihood of death							.32	.61							.45	.60
Knife-related*Primed responsibility							.66	.81							−.42	.80
R^2^	.01		.22		.22		.31		.03		.49		.50		.55	

unstandardised coefficients, ***p < .001, **p < .01, *p < .05.

Lastly, to test **H3**, we explored whether priming participants to consider the consequences of carrying a knife for themselves and others would overcome the potential for exposure to the knife-related images to backfire (although, as above, viewing the knife-related campaign images had no effect on these outcomes). Message prime condition was added in Model 4. As shown in Table 1, there was no main effect of message prime, and no significant interactions between this variable and the other two variables in the model.

## Study 1: Summary

In Study 1, we tested – with a young adult male sample from the general population – whether exposure to fear-based anti-knife carrying campaign images would increase mortality salience and shift respondents’ willingness to carry a knife and the perceived benefits of knife-carrying. We found that although mortality salience increased after exposure to knife-related images, there was no effect of campaign condition on willingness to carry a knife or on perceived benefits of knife-carrying. In other words, viewing knife-related campaign images neither decreased nor increased participants’ attitudes towards knife carrying. However, it could be that the sample used in this study is not reflective of the types of people usually targeted by anti-knife crime media campaigns. Indeed, knife-related self-esteem/cultural worldviews were very low among the sample (mean item score was 1.58 on a 1 to 5 scale). TMT suggests that mortality salience will only increase intentions to engage in risky behaviour for those who have high levels of self-esteem/cultural worldviews related to the risky behaviour (e.g. knife carrying). In Study 2, we replicate Study 1 with a prima facie more appropriate sample: 14–18 years old males recruited primarily via Youth Justice Services and diversionary schemes.

## Study 2: Method

### Participants

Contact was made with a number of youth organisations such as Youth Justice Services, youth groups, Youth Organizations in Uniform (e.g. the Police Cadets, which is now positioned in part as a scheme to divert young people away from offending) and schools, who were asked to help recruit young people to complete the survey. Although the sample size in the study is small (due to ethical constraints, recruitment and accessibility issues particularly around requiring parental consent for every participant), we decided it was more important to access the target audience to test the theory rather than to gain statistical power. Due to ethical constraints, we were not able to ascertain how individual respondents in the dataset were recruited.

In total, we recruited 57 young people aged between 14 and 18 years old, with an even spread across all five age points (ranging from 12% to 26%) and gender (males = 52%). Half of the participants reported their ethnic group to be White – British, White – Irish or any other white background (50%), a fifth reported their ethnic group to be Black or Black British (20.6%), 10.3% were Asian or Asian British, 6.9% were Mixed and 12% were other. There were no significant differences in demographics across the experimental conditions.

### Procedure and Measures

The basic procedure of Study 2 mirrored that of Study 1. The measures used were also identical; however, due to sample size constraints it was not possible to use confirmatory factor analysis to derive and validate latent variables for analysis. Instead, average scores for each scale were used. Cronbach’s Alpha reliability scores indicated good internal consistency across all scales: willingness to carry a knife (α = .87); perceived benefits of carrying a knife (α = .87); and self-esteem/cultural world views (α = .92). See the Appendix Table for full question wordings. Due to ethical constraints, we were unable to ascertain participants’ exposure to knife crime in this study.

## Study 2: Results

### Mortality Salience

As Study 1, an independent samples t-test revealed that our mortality salience manipulation was successful. Consistent with **H1**, participants in the knife-related campaign condition completed significantly more word fragments with death-related words (M = 2.03, *SD* = 1.05) than those in the control condition (M = 1.27, *SD* = 0.83), *t* (55) = −3.01, *p* = .004.

### Perceptions of Knife-Carrying

We conducted the same sequential multiple regression analysis as Study 1 to test H2 and H3. As before, campaign condition was entered as the explanatory variable in Model 1. Knife-related self-esteem was entered in Model 2, and an interaction between the two variables in Model 3. In Model 4, message prime condition was added to the model and a three-way interaction was tested.

As shown in Table 1, inconsistent with **H2a** but consistent with Study 1, there was no significant effect of campaign condition on either willingness to carry a knife (B = -0.14, *p* = .520) or perceived benefits of knife-carrying (B = -0.32, *p* = .220). Knife-related self-esteem scores, on the other hand, were significantly and strongly related to both willingness to carry a knife (B = 0.41, *p* < .001) and perceived benefits of knife-carrying (B = 0.74, *p* < .001). As in Study 1, there was no significant interaction between campaign condition and knife-related self-esteem/cultural worldviews (willingness to carry a knife B = 0.06, *p* = .808; perceived benefits of knife-carrying B = 0.12, *p* = .639). Thus, inconsistent with **H2b**, knife-related self-esteem/cultural worldviews did not moderate the effect of campaign condition on willingness to carry a knife/perceived benefits of knife-carrying. Lastly, we found no evidence in support of **H3**. There was no main effect of message prime condition, and no interactions between this variable and the other two variables in the model.

## Study 2: Summary

Study 2 replicated Study 1, but the sample included 14–18 years old males recruited via Youth Justice Services and diversionary schemes. Although we reasoned this sample would be more appropriate for testing our hypotheses, levels of knife-related self-esteem/cultural worldviews were also low across the sample (mean item score was 1.71 on a 1 to 5 scale), and we found identical results to Study 1. Although mortality salience increased after exposure to knife-related images, there was no effect of campaign condition on willingness to carry a knife or on perceived benefits of knife-carrying. There was no moderating effect of knife-related self-esteem/cultural worldviews and no effect of priming participants’ to consider the consequences of knife carrying.

## Discussion

Knife crime is a serious legal, societal and public health issue. In recent years, anti-knife crime media campaigns have been used as a potential remedy to the knife crime problem. Social media has enabled these campaigns to have widespread reach. Anti-knife crime campaigns often aim to provoke fear by highlighting the potentially devastating consequences associated with carrying or using knives, with the aim of deterring young people from engaging in such behaviour. However, there is little evidence on whether these types of fear appeals are effective. Drawing on TMT, and using samples of young males, the current paper sought to address this gap.

Across both studies, consistent with **H1**, viewing knife-related campaign imagery significantly increased mortality salience. In other words, death-related concepts were more accessible to participants after viewing knife imagery. This finding fits with the broad motivation behind fear based anti-knife crime campaigns: to encourage people to think about the serious and often deadly consequences of using or carrying knives. However, despite mortality being made salient, exposure to knife-related campaign imagery had no impact on participants’ willingness to carry a knife or on the perceived benefits of knife-carrying. Participants in the experimental condition were no more or less likely to report behavioural intentions to carry a knife than participants in the control condition. Thus, **H2a** – that, based on a broadly rational choice perspective, viewing knife-based images would *decrease* willingness to carry a knife and perceived benefits of knife-carrying – was not supported. Making participants more aware of the dangers inherent in carrying a knife did not seem to shift their calculus of risk.

This is of course counter to the rationale for many anti-knife crime campaigns (e.g. ‘Lives not Knives’, Leicestershire Police, 2020; ‘#Lifeorknife”’, West Midlands Police, 2019), which raises questions about the appropriateness of showing graphic and potentially disturbing images to young people. Research has found persuasive evidence that widespread media coverage of traumatic images may have harmful effects on mental health in the long term. In a study by Silver et al. (2013), such media exposure resulted in a stress response that triggered various physiological processes associated with increased health problems over time. Their results suggest that exposure to graphic media images may be an important mechanism through which the impact of collective trauma is dispersed widely. In a time where social media is prominent and often filled with potentially traumatic images, the implications of exposure to such images are currently unknown, but could be damaging. This is especially pertinent with the rise in youth mental health concerns (World Health Organisation, 2020): concerns that are exacerbated further in the current climate of a global COVID-19 pandemic (Youngminds, 2020). Although we did not measure the effect of exposure to knife-related images on participants’ mental wellbeing in our studies, understanding the short and long-term mental health effects of exposure to fear based anti-knife crime campaigns should be an avenue of future enquiry.

We also had a competing hypothesis. **H2b** drew on TMT to suggest that viewing knife-based images will *increase* willingness to carry a knife and perceived benefits of knife-carrying for those who have high levels of knife-related self-esteem/cultural worldviews. We did not find support for this hypothesis either. Although knife-related self-esteem/cultural worldviews strongly predicted behavioural intentions to carry a knife, this did not moderate the relationship between exposure to knife-related imagery and knife-carrying intentions. This finding is in contrast to the predictions of TMT, and previous research showing that people with high behaviour-specific self-esteem are more likely to engage in risky behaviour after having their mortality made salient to them (Jessop et al., 2008; Taubman Ben-Ari et al., 1999). One explanation for this finding is that the act of carrying a weapon was not part of our samples’ socially constructed beliefs or group norms (Arrowood & Cox, 2020). Although we attempted to address this issue in Study 2 by recruiting ‘justice-involved’ youth participants via Youth Justice Services and other avenues, due to ethical constraints we do not know the precise make up of our sample (nor of course how honest participants were about their knife carrying behaviours), across both samples knife-related self-esteem/cultural worldview scores were low. Future work should focus on recruiting individuals with high knife carrying self-esteem/cultural worldviews. Lastly, we found no support for **H3**: priming participants to consider the consequences of carrying a knife on themselves and others had no moderating effect on exposure to knife crime imagery. Again, repeating this study with a more appropriate sample might garner different results.

### Limitations

There are a number of limitations with the current research. The first relates to the nature of our samples. In Study 1, we used Prolific Academic, an online participant recruitment platform, to recruit young adult males. By the very nature of the platform, participants are self-selecting. Although participants were not explicitly informed the study was about knife crime, they were told the study was about social media crime campaigns. One must question how likely it is that an individual who engages in knife-carrying behaviour would also self-select to answer a survey about crime, or indeed sign up to a social research platform in general. This recruitment method therefore likely targeted generally ‘law-abiding’ individuals, and our results reflect this. As already mentioned above, in Study 2 we recruited male youth participants through a variety of means. However, due to ethical constraints, we do not know how many participants from each organisation were recruited. Based on the dates participants completed the study, and the timings of when different organisations were asked to be involved, we can sumise that a large proportion of our sample was likely made up of Police Cadets. These may be individuals who would not normally consider carrying a knife (although it must be noted that involvement in Youth Cadets is often offered to youths who are, or are at risk of becoming, ‘justice-involved’). Moreover, based on the timings we know that at least some participants completed the study when the Youth Offending Services across the UK were recruiting for us.

There are also the typical concerns about reliability, generalisability and validity as a result of using a non-probability convenience sample recruited from a crowdsourcing platform (young adults) and targeted organisations (youths). Additionally, by virtue of the nature of the research, experimental conditions and fictional messages cannot fully replicate real instances of people viewing social media campaigns, as influential factors relating to the context, timing and situation were not fully accounted for here. Future investigation should explore these topics from a more robust methodological perspective.

Finally, the context of the current study is unusual in international terms. The UK is WEIRD (Western, Educated, Industrialised, Rich and Democratic); it also has one of the lowest rates of firearm ownership in the developed world (van Kesteren, 2014). The current policy/political focus on knife crime in the UK may both overstate the level of violence in the country and lack similarity with other countries where firearms are more readily available (and where, in the case of the US and perhaps elsewhere, the incentive structures surrounding weapon carrying are rather different). However, there is no a priori reason to suggest that the basic (hypothesised) psychological mechanisms under-pinning anti-violence campaigns based on fear appeals should be different in the UK to anywhere else, at least in the WEIRD world. It is notable that the results presented in this paper seem to concur with other studies of fear-based crime reduction campaigns, often drawing on an international evidence base, that suggest null effects are often the best that can be hoped for, and that backfire effects are distinctly possible (Lennon et al., 2010; Petrosino et al., 2013; Taubman Ben-Ari et al., 2000).

## Conclusion

Despite the high-profile nature of the problem of knife crime, there is a lack of empirical research on how to tackle it, particularly around how to implement effective campaigns. Fear appeals are often used in anti-knife crime media campaigns in a bid to deter young people from using or carrying knives, yet TMT suggests exposure to fear-based media campaigns could actually have the opposite effect. Our (null) findings have implications both for anti-knife campaigners and those with an interest in TMT.

Considering the implications for anti-knife campaigns, the images we used in this study did trigger respondents to think more about death – which we assume was the original intention of those who created them – but this was not linked to their behavioural intentions. As noted, there is a potential issue here with pushing images on social media, for mass consumption by people of all ages, which may frighten and disturb them for little or no discernible effect. But perhaps the real lesson for anti-knife campaigners is simply that negative messaging may be ineffective, at least in as much as it is disseminated in a more or less untargeted manner. Whether the proposed causal mechanism is rational choice theory or something else, it does not seem that exposing young people to images of knife crime has much of any effect on their views and intentions, at least in the aggregate.

As alternatives to fear-based campaigns we would suggest, first, efforts based more clearly on promoting the types of pro-social attitudes and behaviours – ‘we’ and ‘us’ orientations, rather than ‘I’ or ‘me’ – which proved so important in generating widespread public compliance with COVID-19 restrictions (Bonell et al., 2020; Drury et al., 2021; Jackson & Bradford, 2021). While the ‘pro-social’ prime in the current study did not have the expected effect, stronger and better targeted efforts may, and there is a strong evidence base for the importance of such motivations for behaviour.

Second, police organisations and other legal actors involved in anti-violence campaigns should attend more closely to their relationships with those they are targeting. A range of studies have shown that attitudes toward and propensities to use violence are strongly correlated with trust in legal authorities, most particularly the police (Brennan, 2019; Gau & Brunson, 2015; Jackson et al. 2013; Nivette, 2016). The subjective need, in contexts of low trust, for ‘self-help’ to protect from violence is a key theme of this research, which again stresses the need for developing positive messages and relations with target groups, this time revolving around the willingness and ability of police and other authorities to intervene positively on their behalf. This would seem both an ethically preferable, and potentially more effective, campaign avenue for those seeking to communicate with young people about their potential knife carrying.

Turning to implications for those with an interest in TMT, our findings would seem to be evidence against the theory. However, we suspect they are due more to the nature of our samples, which are unlikely to have contained many individuals for whom carrying a knife was socially valorised. Relevant to the discussion above, one implication here is that if TMT *does* hold, and if our samples *had* contained large numbers of such individuals then a backfire effect may have been forthcoming. Although this remains an unanswered empirical question, it opens up the possibility that while fear-based campaigns targeted at the ‘general’ population may produce no effect on average, and thus at least not be actively harmful, campaigns that are better targeted toward ‘at risk’ groups are *more* likely to produce negative effects. In as much as such groups are more closely aligned with, for example, sub-cultural norms concerning the value of and need for violence, fear-based campaigns could indeed make things worse, not better.

Naturally, it remains a possibility that our findings do count as evidence against TMT. Future research could profitably probe this question, and explore in more depth whether and how TMT can contribute to our understanding of how young people can best be encouraged to avoid carrying knives. Such research could, in particular, consider the interplay between some of the factors outlined above. If fear-based appeals do have backfire effects, as TMT would suggest, is this possibility stronger among those who lack trust in the police and other authorities, for whom ‘self-help’ may be both more appealing and part of a wider sub-cultural orientation toward violence that sees it as inevitable and perhaps desirable? What is the role of multi-faceted group identities, some more ‘pro-social’ than others, in conditioning how people respond to fear-based and indeed any other type of anti-knife messaging? By contrast, if some people are persuaded, or deterred, by fear, as some health research suggests they can be, what characteristics make them ‘immune’ from backfire effects of the kinds highlighted in the TMT literature? What is the difference between (successful) health-related campaigns and the (apparently unsuccessful) efforts considered in the current study? The experiments presented here have only scratched the surface of these issues, but as multiple actors continue with a diverse array of anti-knife crime initiatives based on publicity and communication, there is a pressing need to address them.

## Supplemental Material

sj-pdf-1-jiv-10.1177_08862605211064237 – Supplymenatal material for Fear Appeals in Anti-Knife Carrying Campaigns: Successful or Counter-Productive? Click here for additional data file. Suppemental material, sj-pdf-1-jiv-10.1177_08862605211064237 for Fear Appeals in Anti-Knife Carrying Campaigns: Successful or Counter-Productive? by Zoë Hobson, Julia A. Yesberg, and Ben Bradford in Journal of Interpersonal Violence.

## Note

1. Note that the cadet scheme run by the force included in the study is specifically run, as, in part, a diversion scheme for young people who are risk of crime and offending.

## Appendix A

### Factor Loadings and Model Fit for Confirmatory Factor Analysis, Study 1

**Table:**

	Factor Loadings
Willingness to Carry a Knife	
I Would consider carrying a knife when I leave the house	0.845
There are certain situations when I would consider carrying a knife	0.869
I Can understand why some people would carry a knife	0.678
Self-esteem/cultural worldviews	
*Self-esteem*	
Carrying a knife would make me feel protected when in public	0.840
Carrying a knife would make me feel more positive about myself	0.867
Carrying a knife would give me more confidence	0.905
Carrying a knife would make people respect me more	0.821
*Cultural worldviews*	
My friends would have a higher opinion of me if I carried a knife	0.907
I have more to gain by carrying a knife than I do to lose	0.730
People see knife carrying as a sign of strength	0.443
Others would feel safer in my company if I carried a knife	0.825
Perceived benefits of carrying a knife	
A male punches you in the face over a disagreement and he knocks you to the floor then stands over you and goads you to get up and continue fighting [More or less safe if carrying knife]	0.730
You are mugged when leaving the cinema at night and your wallet is taken. During the mugging you have a struggle with the mugger and get pushed about [more or less safe if carrying knife]	0.796
A local gang who have a reputation for carrying knives have begun to hang around near to your house. You are not friends with them. Some of your friends have been threatened by the gang with knives in the past. [More or less safe if carrying knife]	0.767
You have been at a football match with a group of friends. On the way home, you are confronted by a group of rival fans who start shouting abuse and then attack you and your friends. [More or less safe if carrying knife]	0.721
You and your friends are at the local pub. On the way back from the bar, one of your friends accidentally spills a drink over another group and an argument ensues. The argument escalates and pool cues and glasses are used as weapons. [More or less likely to carry a knife]	0.694
You are due to attend a party with a group of friends. One of them tells you that there is a chance there may be some gatecrashers there after the party was advertised on social media. They tell you that last time there was a party there one of their friends was stabbed after trouble erupted with uninvited guests. [More or less likely to carry a knife]	0.692

Fit indices *χ*^2^(116) = 364.30, *p* < .001; RMSEA = 0.07 [.06, .08]; CFI = 0.97; TLI = 0.97.
