# Supplementary material for: Fear Appeals in Anti-Knife Carrying Campaigns: Successful or Counter-Productive?
Source: J Interpers Violence. 2022 Jan 17;37(23-24):NP21573–98. doi: 10.1177/08862605211064237 (PMC9679559; doi:10.1177/08862605211064237)
Supplement: sj-pdf-1-jiv-10.1177_08862605211064237 – Supplymenatal material for Fear Appeals in Anti-Knife Carrying Campaigns: Successful or Counter-Productive? [file sj-pdf-1-jiv-10.1177_08862605211064237.pdf]

Tweet

West Yorkshire Police
@WestYorksPolice

Work to tackle knife crime is always ongoing and daily work is done throughout the communities of West Yorkshire – particularly in schools to educate young people about the risks of carrying a knife.

Find out more at: westyorkshire.police.uk/news-appeals/k...
#StopKnifeCrime

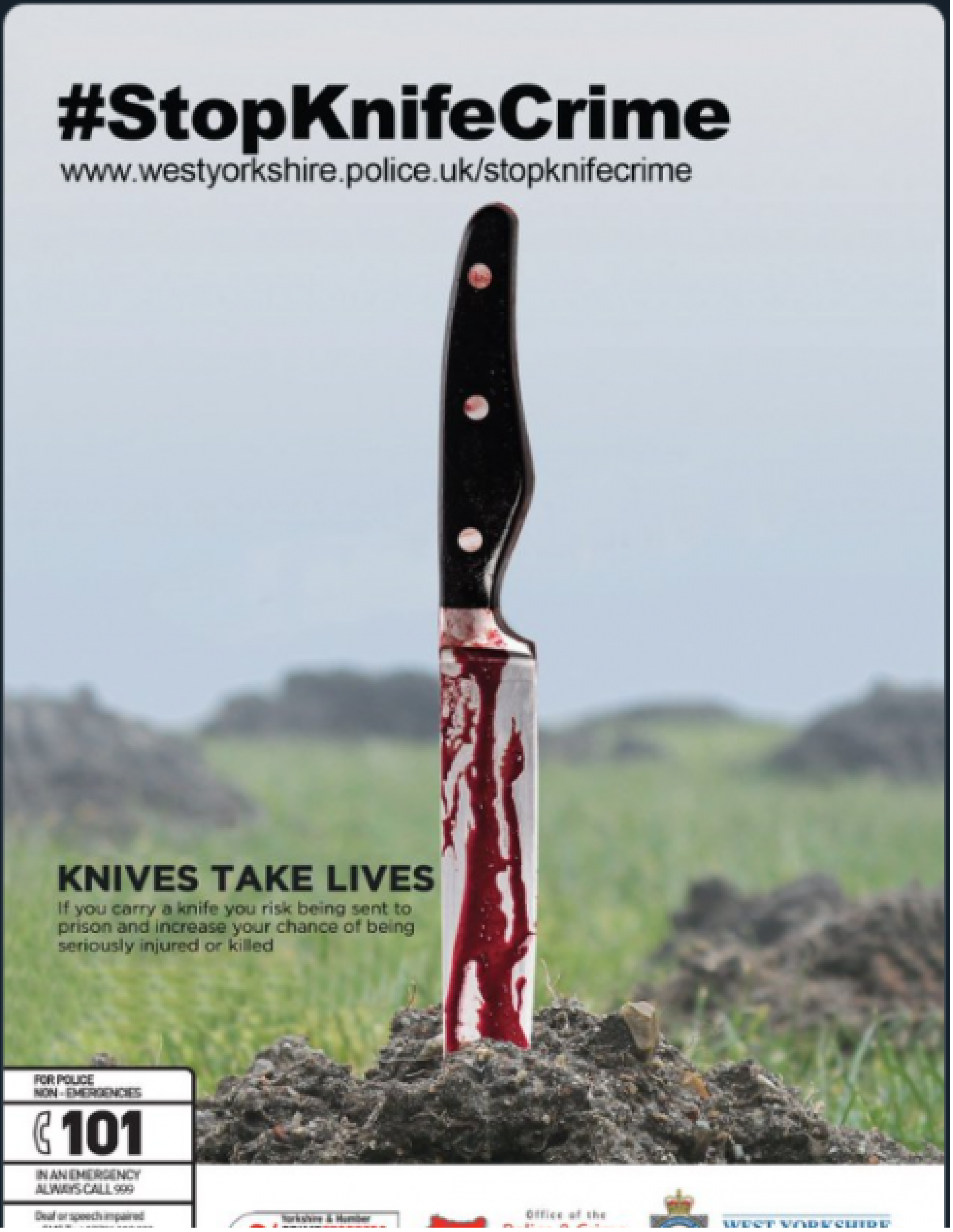

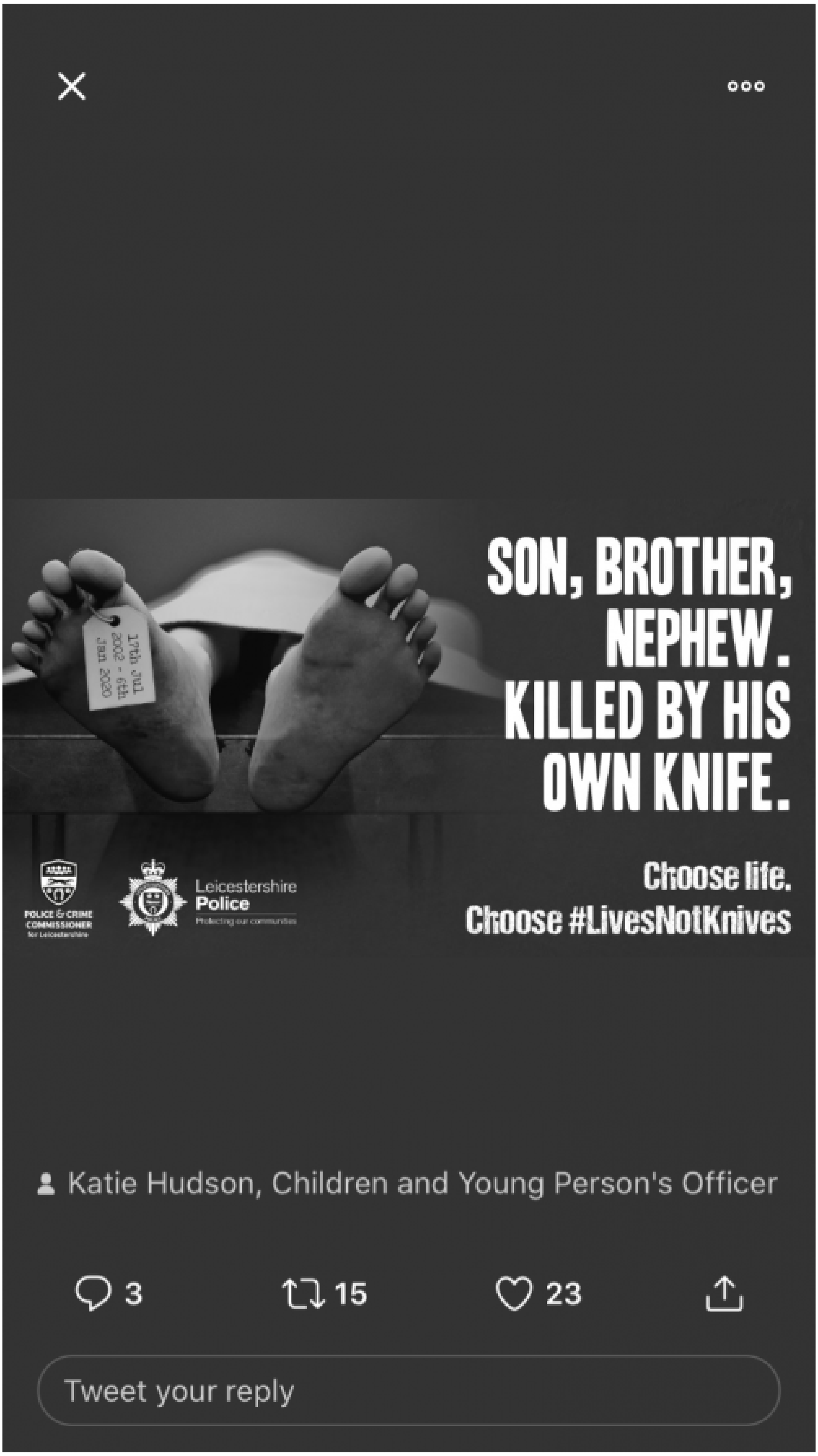
17th Jul 2002 - 6th Jan 2020
SON, BROTHER, NEPHEW. KILLED BY HIS OWN KNIFE.
Choose life.
Choose #LivesNotKnives
POLICE & CRIME COMMISSIONER for Leicestershire
Leicestershire Police
Protecting our communities
Katie Hudson, Children and Young Person's Officer
3
15
23
Tweet your reply

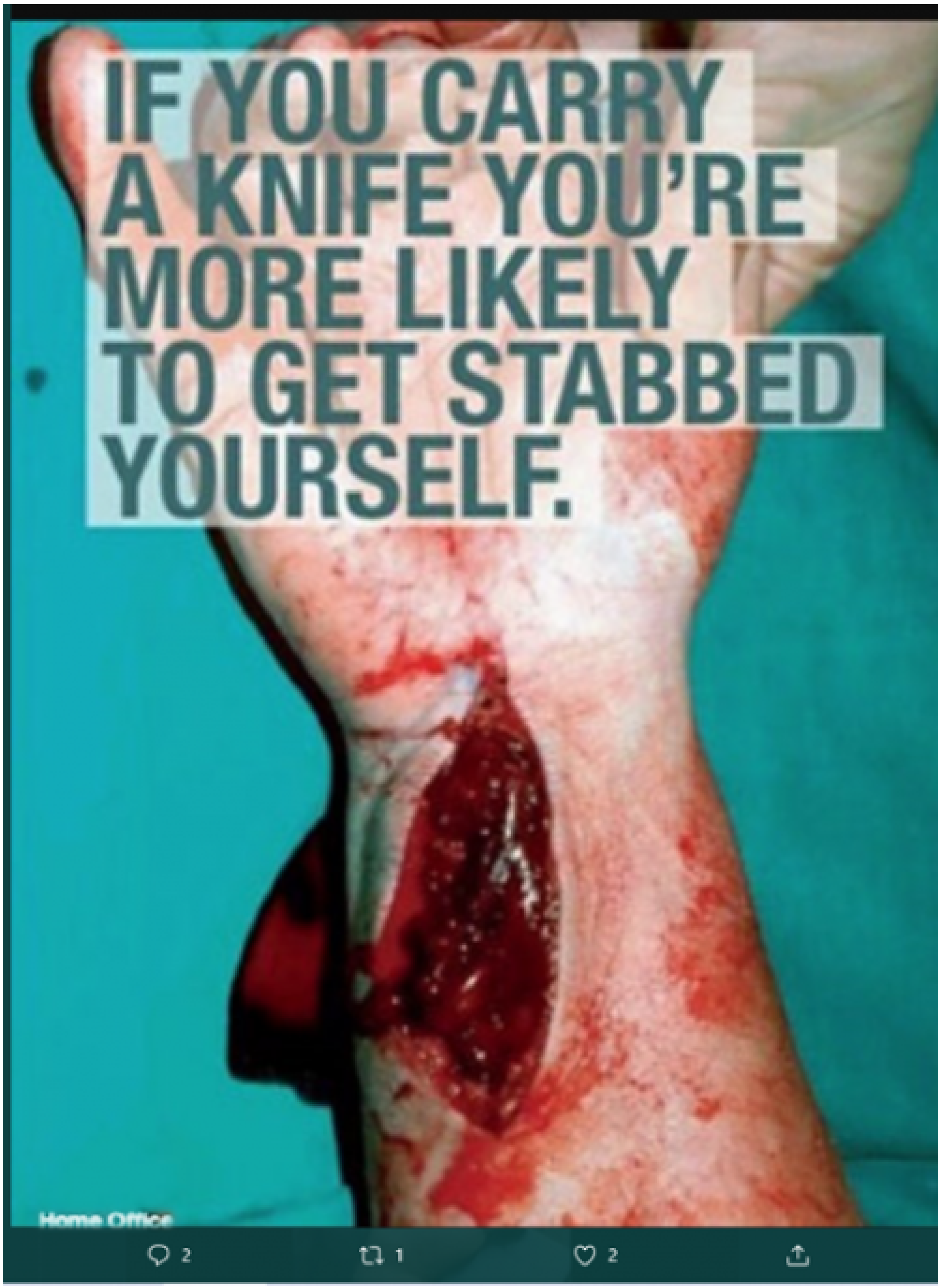
IF YOU CARRY
A KNIFE YOU'RE
MORE LIKELY
TO GET STABBED
YOURSELF.
Home Office
2
1
2

Notts Knife Crime @NottsKnifeCrime · 14 Mar 2019

Those carrying knives are statistically far more likely to be victims themselves of knife crime – often with their own knife they were carrying. Speak to your family members if you have concerns about them carrying a knife
#LivesNotKnives #opsceptre

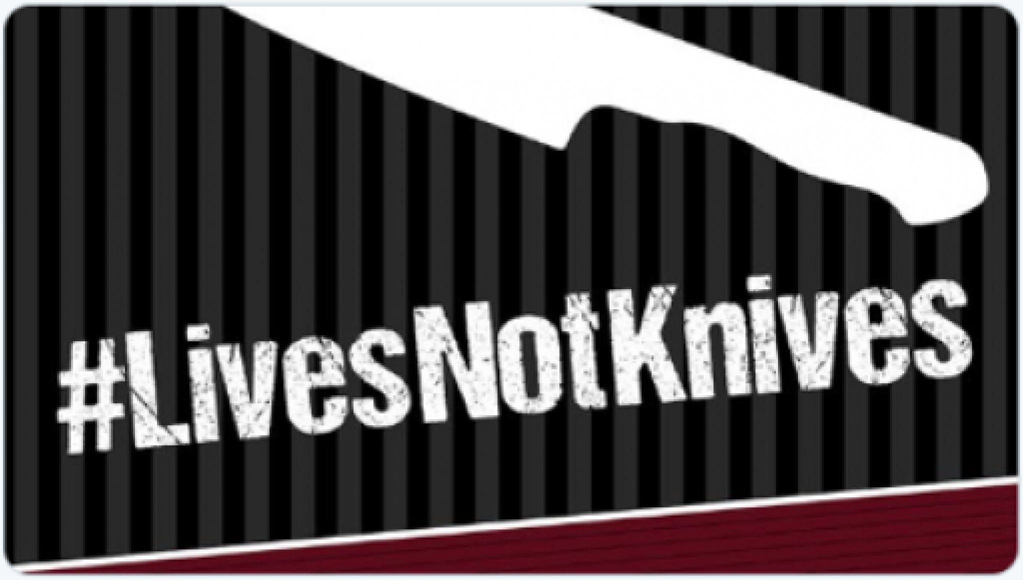

3 3

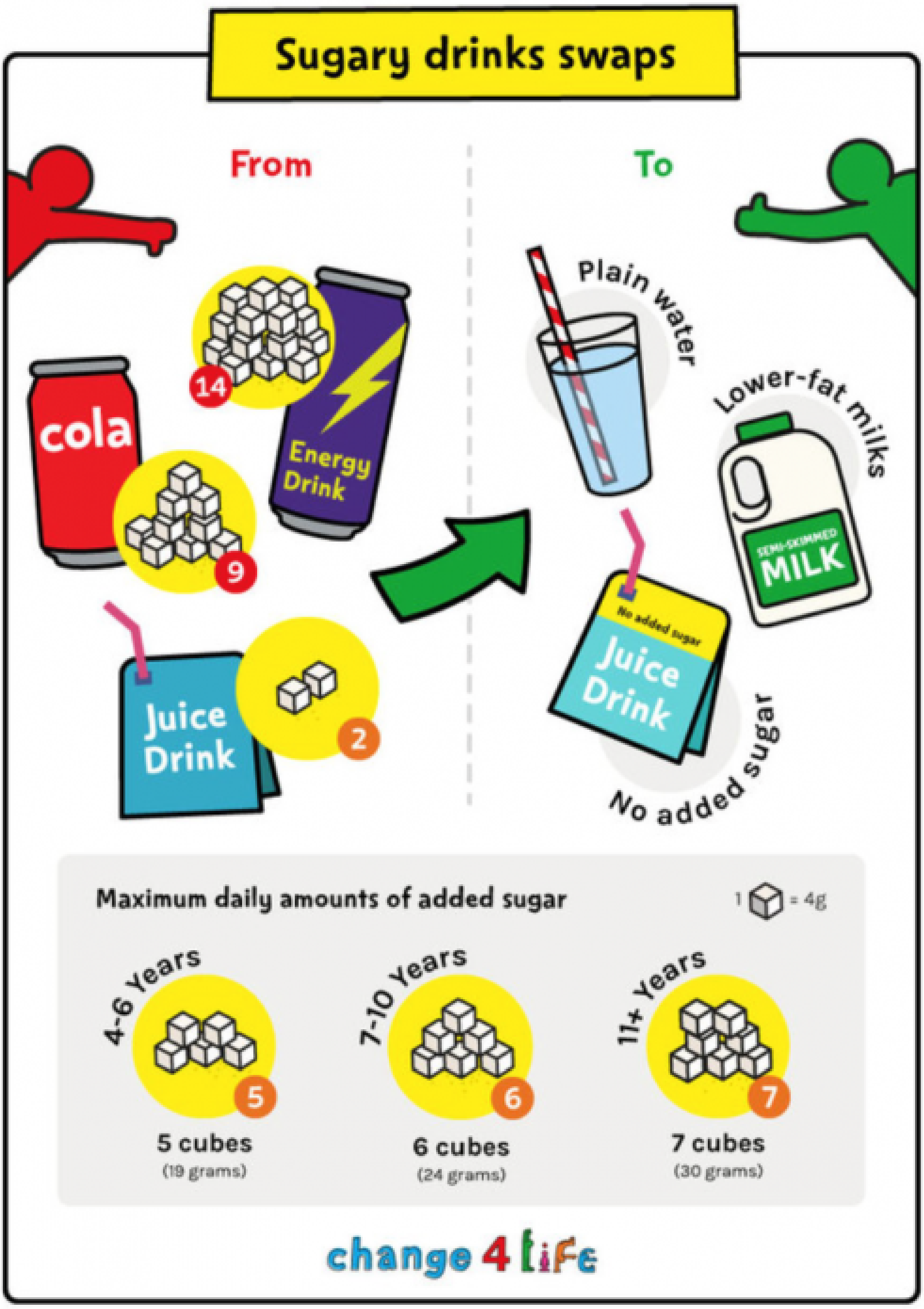
Sugary drinks swaps
From
To
cola
Energy Drink
14
9
Juice Drink
2
Plain water
Lower-fat milks
SEMI-SKIMMED MILK
No added sugar
Juice Drink
No added sugar
Maximum daily amounts of added sugar
1 = 4g
4-6 Years
5
5 cubes
(19 grams)
7-10 Years
6
6 cubes
(24 grams)
11+ Years
7
7 cubes
(30 grams)
change 4 life

West Midlands Police
@WMPolice

Installing the latest #SoftwareUpdates is a quick and effective way to secure your 📱💻🖥️ 🎮 📺 against cyber criminals.
Have you got the latest updates?

For more info, visit ➡️ ow.ly/K3sR50yqodr

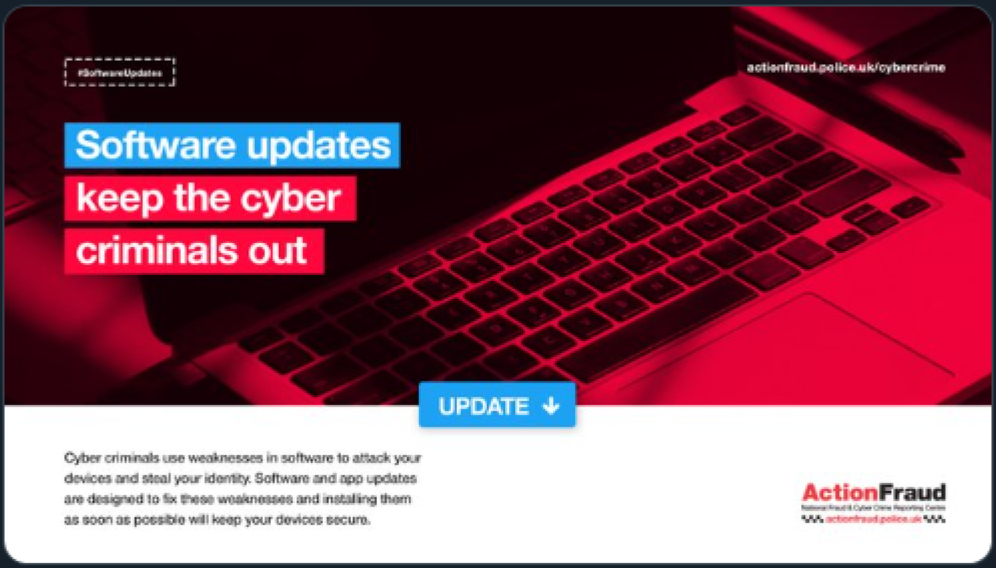

9:01 am · 24 Feb 2020 · Hootsuite Inc.

2 Retweets **6** Likes

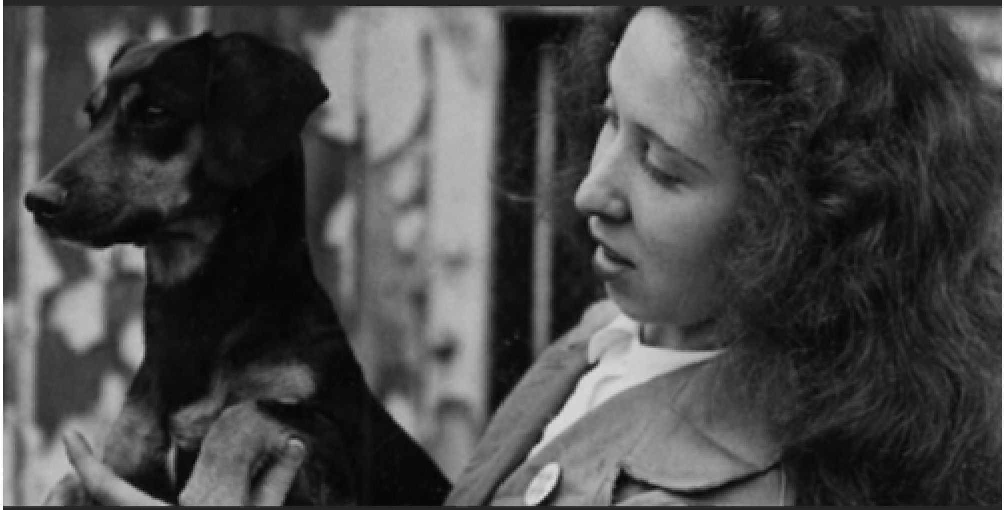

Blue Cross @The_Blue_Cross · 5d

To help servicemen bring dogs back from overseas, our boarding kennels in Blackheath, London, were used for quarantine purposes.

...

6 24

Tweet your reply

Tweet

DVLA
@DVLAgovuk

❗ We're clamping untaxed vehicles in #London and #Leicester today. If your vehicle isn't taxed, do it now: ⤵️

↪️ gov.uk/dvla/taxyourve...

#TaxItOrLoseIt

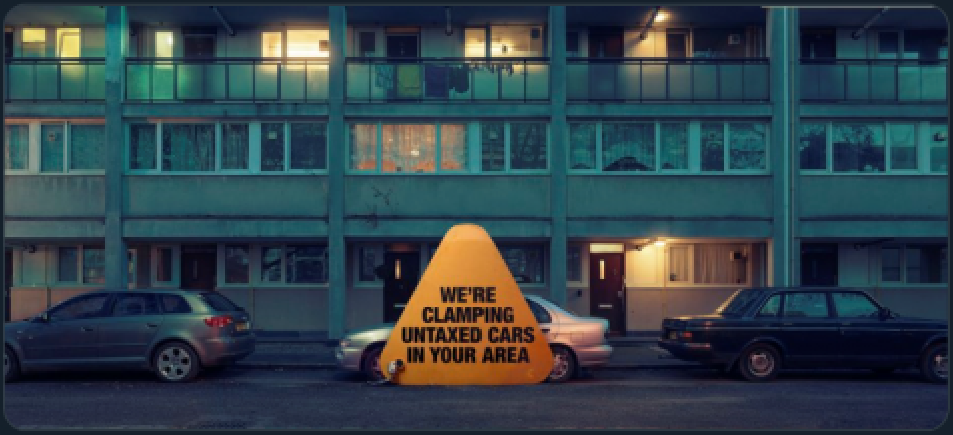

4:17 PM · Feb 19, 2020 · Salesforce - Social Studio

CARRYING A
KNIFE
HAS CONSEQUENCES

Carrying a knife can result in your own death.

You are 3 times more likely to be stabbed if you go out carrying a knife.

CRIMESTOPPERS 0800 555 111

CARRYING A
KNIFE
HAS CONSEQUENCES

Carrying a knife can have devastating consequences on your friends and family.

No parent or grandparent would ever want to see their child get injured or be killed.

CRIMESTOPPERS 0800 555 111

101
